# Obituaries

**Published:** 1888-11

**Authors:** 


### Obituaries.

The Death of Dr. Harvey Jewett, of Canandaigua, which occurred at his home September 4, 1888, removes one of the strongest medical characters in Central New York from his earthly field of usefulness. Dr. Jewett has been so long identified with the best interests of the profession, and has done so much to advance them, that his loss will be seriously felt for a long time; nor is it probable that any one will ever quite fill the vacancy that death has thus created. Dr. Jewett had practiced more than fifty years, grown up and been identified with almost every public interest and improvement in his region, been active in organizing and maintaining all medical societies—local, state and national—that were worthy of aid and support—for these and other reasons his death causes more than a local grief; it is well-nigh universal.

Dr. John S. Lynch, of the faculty of the College of Physicians and Surgeons, Baltimore, died at his home September 27, 1888. Dr. Lynch has been long and favorably known as a teacher and writer, and for his great professional usefulness. He had been twice Vice-President of the American Medical Association, and was an active promoter of its interests.

Dr. Elkanah Williams, of Cincinnati, one of the most accomplished ophthalmologists in this country, died at Hazelwood, Pa., October 5, 1888. For upwards of thirty-two years, Dr. Williams was active as a student, teacher, and writer in his chosen field of specialism.

